# Gamma detector dead time correction using Lambert *W* function

**DOI:** 10.1186/s40658-020-00296-w

**Published:** 2020-05-11

**Authors:** Jan W. T. Heemskerk, Michel Defrise

**Affiliations:** Department of Nuclear Medicine, Universitair Ziekenhuis Brussel, Brussel, Belgium

## Abstract

**Background:**

For therapeutic applications of several isotopes (e.g., ^131^I, ^153^Sm, ^177^Lu) in nuclear medicine, the high activities typically applied require accurate dead time correction in early time point imaging. We present a novel, straightforward dead time correction method using the Lambert *W* function, which is in principle exact for the paralyzable detector model with a single parameter τ (i.e., dead time).

**Results:**

As a proof of concept, the method is validated with a simple model: a commonly used isotope, ^99m^Tc, with a single photopeak. We measured count rates of a gamma camera both intrinsically and extrinsically (i.e., with collimators) with point sources in air and in a scatter phantom (extrinsic only). τ was estimated for both open window (τ_OW_) and a ^99m^Tc photopeak window (τ_Tc_), using a “graphical” method for fitting the count rate of decaying sources. These values for τ were subsequently used for dead time correction.

τ varied significantly between the different geometries for both energy windows, but τ_OW_ was more reproducible than τ_Tc_, particularly for the scatter phantom measurements.

τ_OW_ measured from the phantom measurements was approximately 30% lower than τ_OW_ from the intrinsic measurement but corresponded within 15% with the extrinsic point source measurements. Accordingly, using the intrinsic τ_OW_ led to an overcorrection of 8% at high count rates; τ_OW_ from the extrinsic point source measurements corrected the phantom measurement to within 2%.

However, significant differences were observed between τ_Tc_ values. All measured τ_Tc_ values underestimated dead time losses in a second independent phantom measurement, with even τ_Tc_ from the first phantom measurement underestimating activity with 5–10% at the highest count rates. Based on measurements of the effect of energy window settings and geometry, we tentatively attribute the added dead time losses to pulse pile-up.

**Conclusions:**

Analytic dead time correction based on the Lambert *W* function is accurate for the range in which gamma detectors behave as paralyzable systems. However, further investigation indicated measured τ values to be variable with geometry as well as window fraction. We propose that dead time correction should be based on the open window value, τ_OW_, corrected for window fraction.

## Background

### Quantitative imaging

For quantification of nuclear medicine images, the number of detected counts within a region of interest (ROI) or image is assumed to be proportional to the activity *A* within the (imaged) region.

As stated in MIRD pamphlet 16 [1], the system calibration factor *C* (count rate per unit activity) can be obtained by counting a known activity for a fixed period of time within a standardized geometry in air relative to the scintillation camera using designated camera acquisition settings:

1 $$ {R}_{t,\mathrm{EW}}={C}_{\mathrm{EW}}\bullet A/\left(F\bullet {e}^{\mu_ed}\right), $$

with *C*_EW_ the sensitivity and *R*_*t,*EW_ the count rate for a chosen energy window EW, and *A* the activity within the imaged region of interest. The factor ${e}^{\mu_ed}$ is the correction for absorption of radiation within the object, and *F* is a factor that corrects for background due to activity in overlying and underlying tissue.^1^ The subscript *t* in *R*_*t*,EW_ indicates the true count rate here, as Eq. (1) neglects the effect of dead time.

For a point source in air, with negligible absorption and background, the denominator in Eq. (1) can be ignored, as it is unity. This results in the count rate *R*_*t*,EW_ being proportional to activity *A* in the imaged region, with proportionality constant or linear response coefficient [2], $$ {C}_{\mathrm{EW}}=\raisebox{1ex}{${R}_{t,\mathrm{EW}}$}\!\left/ \!\raisebox{-1ex}{$A$}\right. $$.

However, for high count rates Eq. (1) is no longer valid because of dead time losses: the inability of a camera to detect a second scintillation event within a certain resolving or dead time, τ, after a previous event.

With the possible exception of very specific procedures such as first pass cardiac imaging, count rates rarely suffer significant losses during diagnostic nuclear medicine imaging procedures. However, for several radioisotopes, count rates can increase significantly (∼250 kcps in an open energy window), during imaging performed after internal radionuclide therapies. This can lead to substantial count rates losses [3], in a context where quantification is arguably more important.

In order to accurately determine the activity from the counts in the image, therefore, correction for dead time losses is an absolute necessity. In clinical radioimmunotherapy trials with ^131^I-labeled hapten, a small molecule that stimulates the production of antibodies, Ferrer et al. [4] have obtained correction factors of over 200% for activity in organs with large uptake. Similarly, Uribe et al. [5] have obtained dead time correction factors of up to 23% and 20%, resp., for the 113 keV and 208 keV photopeaks of ^177^Lu in phantom experiments designed to emulate lutetium targeted radiotherapy (TRT).

A number of authors have developed theoretical descriptions of dead time [3, 6, 7], have investigated dead time effects on image quality and quantification [4, 8], or have presented different correction methods [5, 9–11]. However, even though very recently an analogous method has been presented for dead time correction in pixel detectors [12], previously, no closed form method for dead time correction in nuclear medicine imaging has been presented.

### The dead time effect of a paralyzable system

Modern gamma cameras have been demonstrated to behave as paralyzable systems, see, e.g., Silosky et al. [3] and Guy et al. [9].

For such paralyzable detectors, an event will not be recorded when it is incident on the detector within an interval smaller than the resolving time, τ, after a previous event. Furthermore, if an event occurs within this interval, the dead time is extended by another period τ. To simplify notations, the subscript EW indicating the dependence of the count rate, the dead time parameter, and the sensitivity on the selected energy window will be omitted below. The impact of the energy window will be discussed in detail below.

For a paralyzable detector, the relationship between actual and observed count rate is described by the well-known equation [8]^2^:

2 $$ R^{\prime }={R}_t{e}^{-{R}_t\bullet \tau }, $$

where *R*′ is the observed count rate, *R*_*t*_ is the actual count rate in absence of dead time losses, and τ is the dead or resolving time, all for a certain energy window. We assume here that the dead time affects the detector as a whole and that therefore *R*′ and *R*_*t*_ denote the total count rate on the detector.

As is well known, the detected count rate, *R*′, reaches a maximum *R*′_max_ = (*e* ∙ *τ*)^−1^ for a true count rate ${R}_t^{\ast }={\tau}^{-1}$. For *R*_*t*_ > τ^−1^, *R*′ decreases monotonically to 0. Therefore, Eq. (2) has two solutions for each *R*′; we are only interested in the solution ${R}_t<{R}_t^{\ast }$.

### Lambert *W* function

To calculate *R*_*t*_ from *R*′, we use the Lambert (or log-product) function, *W*(*z*), which is the inverse of the function *f*(*z*) = *ze*^*z*^ (see, e.g., [13, 14]). Filling in – *R*_t_ · τ for *z*, one can derive an expression for *R*_t_ from *R*′, τ, and Eq. (2):

3 $$ {R}_t=-\frac{W\left(-{R}^{\prime}\bullet \tau \right)}{\tau }. $$

Because we are interested in the real (as opposed to imaginary) solution for ${R}_t<{R}_t^{\ast }$, we only consider the principal branch, *W*_0_, of the Lambert function; its Taylor series around 0 is [13]:

4 $$ {W}_0\left(z^{\prime}\right)={\sum}_{n=1}^{\infty}\frac{{\left(-n\right)}^{n-1}}{n!}z{\prime}^n $$

and converges for *z*′ < *e*^−1^, which is the relevant domain where *R*′ · τ < *R*′_max_ · τ = *e*^−1^.

In practice, including post-therapy imaging, a gamma camera is usually operated well below the maximum count rate. Consequently, it is safe to assume that *R*'τ (or *z*′) is small for the relevant range of *R*′, and the corrected count rate can be accurately estimated by limiting the Taylor series, Eq. (4), to *N* terms. We have used *N* = 10, which is accurate to within 1% for up to 40% dead time loss. Given an accurate estimation of τ, Eq. (3) allows a straightforward calculation of *R*_*t*_ from *R*′.

Combining Eqs. (1) and (4) allows us to estimate the activity, *A*, given measured count rate, *R*′,

5 $$ A=\frac{1}{C\bullet \tau }{\sum}_{n=1}^{\infty}\frac{(n)^{n-1}}{n!}{\left({R}^{\prime}\bullet \tau \right)}^n. $$

Equation (5) requires an accurate determination of *C* and τ, but it allows a closed form correction for dead time losses. In the present paper, the method is applied to planar images; in principle, the method can be readily expanded to whole body or tomographic imaging as well.

### Effect of energy window settings on dead time

The effect of energy window settings on dead time is a further matter of discussion. Several authors have found a large dependence of τ on energy window settings [3, 7, 15]. Cherry et al. [16] state that for *counting* systems, an approximate equation for apparent dead time is τ_EW_ = τ/*w*_f_, where τ_EW_ is the apparent dead time for a certain energy window, τ is the actual dead time per detected event, and *w*_f_ = *R*′_EW_/*R*′ is the window fraction.

Similarly, in the case of a gamma camera, events on the detector can be included or excluded from the image only *after* the deposited energy has been calculated [15]. This implies that when measuring ^99m^Tc photons, one has to consider at least some amount of dead time caused by photons that are not recorded in the image, i.e., scatter or background events. Based on the results of Silosky et al. [3], for SPECT detectors, Uribe et al. [5] generalize the above equation to

6 $$ {\uptau}_{\mathrm{Tc}}={\uptau}_{\mathrm{OW}}/{\left({w}_f\right)}^{\upeta}, $$

where η is a positive constant.

Furthermore, Uribe et al. [5] state that count losses due to electronics are (rather) energy independent. If, correspondingly, the photopeak fraction of a particular measurement is count rate independent, then necessarily, the *fraction* of counts that is lost due to dead time is independent of energy window and therefore *e*^−*R* ∙ *τ*^ is independent of the selected energy window.

From this, it follows that −*R*_*Tc*_ ∙ *τ*_*Tc*_ =  − *R*_*OW*_ ∙ *τ*_*OW*_, and therefore *τ*_*Tc*_ = *τ*_*OW*_/*w*_*f*_, (i.e., η = 1), as above, indicating that dead time losses are determined by a single system dead time parameter τ. However, this simplification does not consider the effects of pulse pile-up, which can reduce the intensity of the photopeaks (i.e., *w*_f_) with increasing count rate [17–19].

To investigate the relationship between energy window settings and measured dead time and whether a single parameter τ can define the dead time behavior of a detector, we measured dead time losses for several energy window settings.

## Methods

### Estimation of dead time τ

Two methods are generally used to determine dead or resolving time, τ: a decay rate method and a dual source method (see, e.g., [3, 16, 20]).

The decay rate method uses a single source of activity and monitors the detected count rate, *R*′, as a function of time as the source decays. Measuring over a large range of count rates (approximately 7 half-lives, or over 40 h for ^99m^Tc), the dead time parameter is estimated by fitting the observed count rates with Eq. (1). Note that for this fit, several authors assume that at low enough count rates dead time has no effect, i.e., *R*′ = *R*_*t*_ [3, 20].

Two alternative methods were used in this paper: first, the dual source method (Huttig [6] and Adams et al. [7]), and second, a variation of the graphical method discussed in Knoll [20]. This method is an improvement of the decaying source method because it fits all measured count rates vs. activity (or time) but makes no assumption on the absence of dead time loss for low count rates.^3^

For the two methods described below, all count rates were corrected for background by simple subtraction.

### Dual source method

For the dual source method, two sources (of similar activity) are measured, first separately and then concurrently in front of the detectors. With *R*′_1_ and *R*′_2_ denoting the observed count rates for sources 1 and 2 separately and *R*′_12_ for the concurrent measurement, τ is estimated from

7 $$ \tau =\frac{2{R}_{12}^{\prime }}{{\left({R}_1^{\prime }+{R}_2^{\prime}\right)}^2}\mathit{\ln}\left(\frac{R_1^{\prime }+{R}_2^{\prime }}{R_{12}^{\prime }}\right). $$

Neglecting errors due to Poisson counting statistics, this estimate is exactly provided the two sources have equal activity. When *R*_*t*,1_ ≠ *R*_*t*,2_ the relative error is of order (*R*_*t*,1_ − *R*_*t*,2_)^2^/(4 (*R*_*t*,1_ + *R*_*t*,2_)^2^) and remains small if the two sources have similar activity.

Silosky et al. [3] show that τ can be accurately estimated for *R*_12_ > 35% of max count rate, but that above a certain count rate (approx. 95% of maximum count rate), the paralyzable detector model (PDM) will no longer be applicable and the estimated τ will be inaccurate as a result. Furthermore, they have verified that τ can be accurately estimated to within 1% when the ratio of single source activity to total activity is between 44% and 55% (i.e., the sources are equal to within 20%).

### Graphical count rate method

For an alternative determination of τ, the data that were measured for the dual source method were additionally fitted for decay and dead time loss. The advantage of this method to the dual source method is that all data points are considered simultaneously, instead of in pairs of three. The implications of this will be further discussed below.

Unlike most other count rate methods, for a graphical fit of count rate vs. activity, no assumption has to be made with regard to the magnitude of dead time loss at low count rates. The method presented here is an adaptation of that illustrated in Knoll [20].

Rewriting Eqs. (1) and (2), one obtains $$ \raisebox{1ex}{$R^{\prime }$}\!\left/ \!\raisebox{-1ex}{$A$}\right.=C\bullet {e}^{-C\bullet A\bullet \tau } $$ , and thus

8 $$ \ln \left(\raisebox{1ex}{$R^{\prime }$}\!\left/ \!\raisebox{-1ex}{$A$}\right.\right)=\ln \left(C\bullet {e}^{-C\bullet A\bullet \tau}\right)=\ln (C)-C\bullet \tau \bullet A. $$

Therefore, if one plots $$ \ln \left(\raisebox{1ex}{$R^{\prime }$}\!\left/ \!\raisebox{-1ex}{$A$}\right.\right) $$ vs. *A*, one gets a linear function with intercept ln(*C*) and slope − *C*τ. We estimate these parameters by an unweighted least squares linear fit.

The same data that were acquired for the dual source method were used for the graphical calculation of τ (and *C*).

### Experimental setup

For the evaluation of the Lambert dead time correction method, count rates were measured over a wide range of activities on a SPECT camera of a principal vendor: a Philips BrightView SPECT camera, with detectors consisting of 59 PMTs reading out a 40.6 × 54 cm^2^, 3/8″ (9.5 mm) thick NaI(Tl) scintillation crystal (approx. 90% detection efficiency at 140 keV). The detectors can be read out with a pixel size down to 0.58 mm (1024 × 1024 matrix). The manufacturer specifies a maximum count rate of 350 kcps, with 20% loss of count rate at 300 kcps.

Data were acquired without (intrinsic) and with low-energy high-resolution (LEHR) collimators (extrinsic).

For practical reasons, we implemented a slight modification of the dual source method, which was replaced by a triple source method, as described below.

### Intrinsic calibration

The intrinsic (i.e., without collimators) measurement was performed according to the setup of the NEMA count rate test, with point sources placed at more than five times the size of the Uniform Field of View (UFOV) from the surface of the detectors, with six 1 mm copper plates shielding the sources, in order to suppress (back-)scattered photons and to create a clean photopeak. The sources were placed at approximately 300 cm from the detectors.

The ^99m^Tc sources used for these measurements had initial activities of 125 MBq (sources 1 and 2) and 250 MBq (source 3). These sources were measured, through decay, down to activities of approx. 4.6 and 9.2 MBq, resp.

Before and after each series of measurements, background measurements were performed of 300 s’ duration; measurements with sources were 60 s. Images were acquired simultaneously for two energy window settings, open window (OW) from 0 to 1022 keV, and ^99m^Tc from 126.5 to 154.6 keV. A series of measurements consisted of measuring different combinations of activities, i.e., all sources individually, both low-activity sources together, and all three sources together.

For both detectors, for both OW and ^99m^Tc energy windows, 17 values of τ were calculated using the dual source method, Eq. (7), from a total of 49 measurements. These data were also used for the graphical method to calculate τ and *C*.

### Extrinsic verification

The goal of the extrinsic measurements was to verify the accuracy of the proposed dead time correction and the consistency between the dead time values measured with and without collimator and with and without scattering medium. The entire extrinsic measurement series (both with and without scattering medium) was performed twice, on two separate days, to have a measure of reproducibility.

The extrinsic measurements comprised a similar experiment as for the intrinsic acquisitions, measuring count rates, *R′*, for varying activity, *A*, with the same energy window settings, but for point sources in air and for sources placed within a NEMA (PET) scatter phantom, using the low-energy high-resolution (LEHR) collimators. Only two sources were used for these measurements, corresponding to the cases 1,2, and 3 in Table 1 (dual source dead time measurement.)

**Table 1:** Overview of combinations of sources and resulting τ values

Measurement	Source(s)	Count rate	
1	1	*R*′_1_	
2	2	*R*′_2_	
3	1 and 2	*R*′_12_	*R*′_1_
4	3		*R*′_2_
5	1, 2, and 3		*R*′_12_
Result		τ at *A*_1 + 2_	τ at *A*_1 + 2 + 3_

The sources that were used for the extrinsic verification measurements had activities of approx. 1.4 GBq at the start of the measurements and 2.8 GBq combined. This combined activity should result in count rates representative of those for patient acquisitions at actual (therapeutic) activities.

Care was taken to place the sources within the NEMA phantom in as reproducible a manner as possible, with both sources close to the center of the phantom. The detectors and the patient bed were maintained at fixed positions, with the detectors 25 cm above and below the center of the phantom.

The verification of the Lambert *W* (or log-product) correction method relies mainly on an investigation of the *linearity* of the count rate vs. activity (i.e., the sensitivity *C*_EW_), because the absolute value of *C*_EW_ is also a function of collimator and the imaged object (through attenuation and scatter).

Finally, for these extrinsic measurements, additional energy windows were measured to investigate the relationship between dead time τ and energy window width. Simultaneous to the open window (0 to 1022 keV) and 20% ^99m^Tc window (126.5 to 154.6 keV), four extra energy windows centered around 85%, 95%, 105%, and 115% of the ^99m^Tc peak were measured, each 14 keV (10% of ^99m^Tc γ energy) wide.

## Results

### Intrinsic measurements

**Fig. 1:**
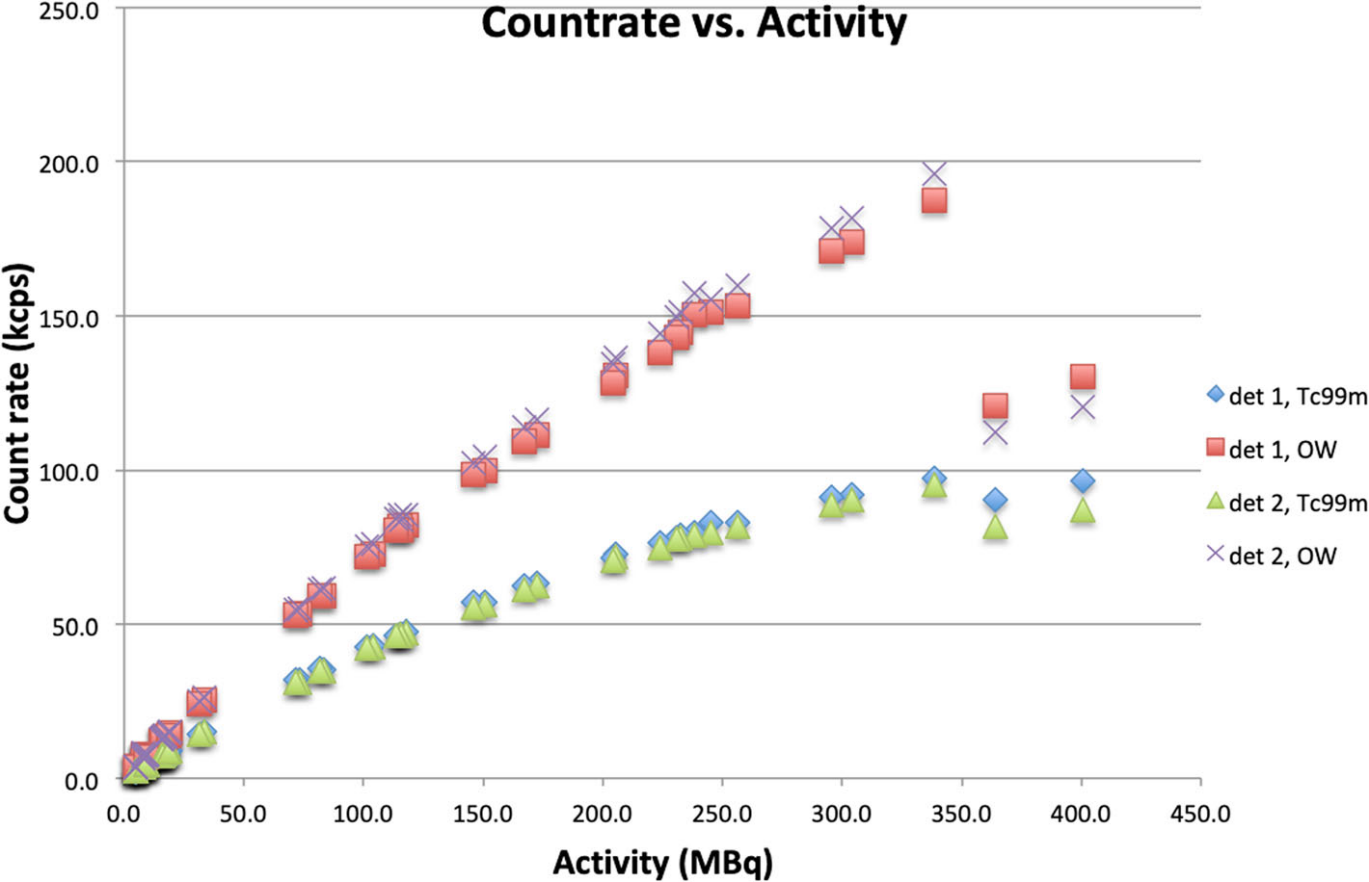
The measured count rate (kcps) vs. activity (MBq) for the intrinsic measurement for both detectors, both OW and ^99m^Tc energy windows. Note the sharp, step-like, decrease in count rate (for both detectors and both energy windows) above approx. 350 MBq

Figure 1 shows the measured count rate (in kcps) against the total activity, for 49 measurements with either one, two, or all three sources. Note that, in accordance with the results of Silosky et al. [3], the paralyzable detector model (PDM) is no longer applicable above a certain count rate (around 200 kcps for the OW at an activity of ± 350 MBq).

### Dual source dead time calculation

**Fig. 2:**
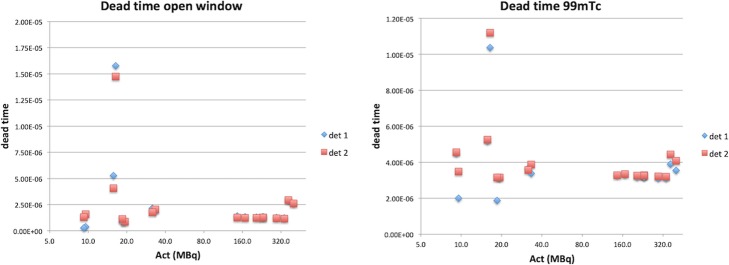
Calculated dead time τ (s) vs. total activity (MBq) for the intrinsic measurement for both detectors for the open window (OW 0–1022 keV, left) and ^99m^Tc (126.5–154.6 keV, right) energy window acquisitions

From each series of measurements, 17 sets of individual and combined sources in total, dead time has been calculated, for both OW and ^99m^Tc energy windows, according to Eq. (7). The results are plotted in Fig. 2.

Figure 2 indicates that the calculated τ values for both the OW and ^99m^Tc energy windows are fairly constant for the range of 30–340 MBq (of total activity). Note that the horizontal axis is logarithmic to better visualize the variability at low count rates.

Table 2 lists the average dead time τ (μs) for the intrinsic measurements over the range of 30–340 MBq. For both detectors, both energy windows, τ is reasonably precise. It must be noted, however, that there may be a slight decrease of the calculated τ with activity over that range. This is not uncommon: Silosky et al. [3] show an increase of measured τ with activity for a camera of another vendor, and Adams et al. [7] show τ either increasing and decreasing with activity, depending on the manufacturer.

**Table 2:** Dead time values (μs) for the intrinsic point source measurements

Detector	Energy window	τ (μs)	
		From dual source method	From graphical method
Det 1	OW	1.31 (0.06)	1.30 (0.04)
	^99m^Tc	3.19 (0.08)	3.16 (0.04)
Det 2	OW	1.23 (0.04)	1.25 (0.03)
	^99m^Tc	3.26 (0.04)	3.32 (0.05)

Dead time values (μs) from the dual source method and the graphical method for the intrinsic point source measurements. Values between brackets are the standard deviation

### Graphical dead time calculation

The same data were used to calculate τ using the graphical method, according to Eq. (8).

**Fig. 3:**
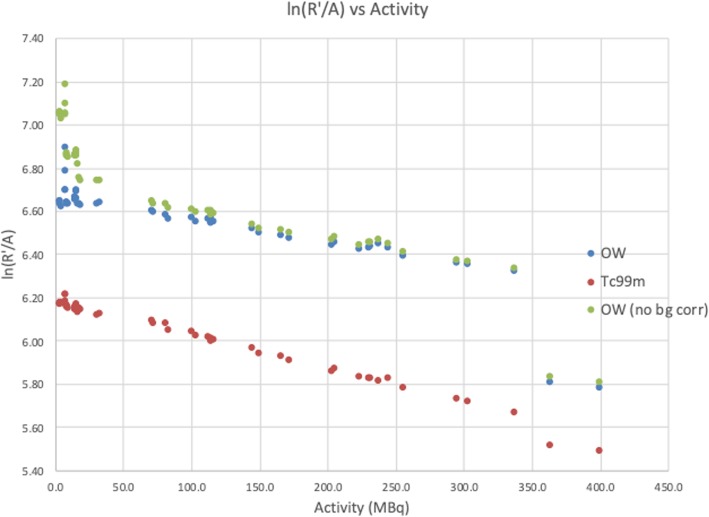
Graphical dead time calculation. Plot of ln(*R′*/*A*) vs. *A* (MBq) for the intrinsic measurement for detector 1 for the OW (blue, green) and ^99m^Tc (red) energy window acquisitions

From Fig. 3 and Eq. (8), we calculated *C*_OW_ and τ_OW_ to be 0.78 kcps/MBq and 1.30 μs, and *C*_Tc_ and τ_Tc_ to be 0.48 kcps/MBq and 3.16 μs. Not correcting the background would result in *C*_OW_ = 0.98 kcps/MBq and τ_OW_ = 2.01 μs for the open window acquisition. Calculated dead time values, τ, are listed in Table 2, and values for *C* in Table 3 below.

**Table 3:** Intrinsic sensitivity *C*_EW_ from dead time corrected count rate measurements

Detector	Energy window	*C* (kcps/MBq)	
		From graphical analysis	From corrected count rates
Det 1	OW	0.78 (0.003)	0.79 (0.037)
	^99m^Tc	0.48 (0.002)	0.48 (0.007)
Det 2	OW	0.82 (0.003)	0.82 (0.036)
	^99m^Tc	0.48 (0.001)	0.48 (0.008)

**Fig. 4:**
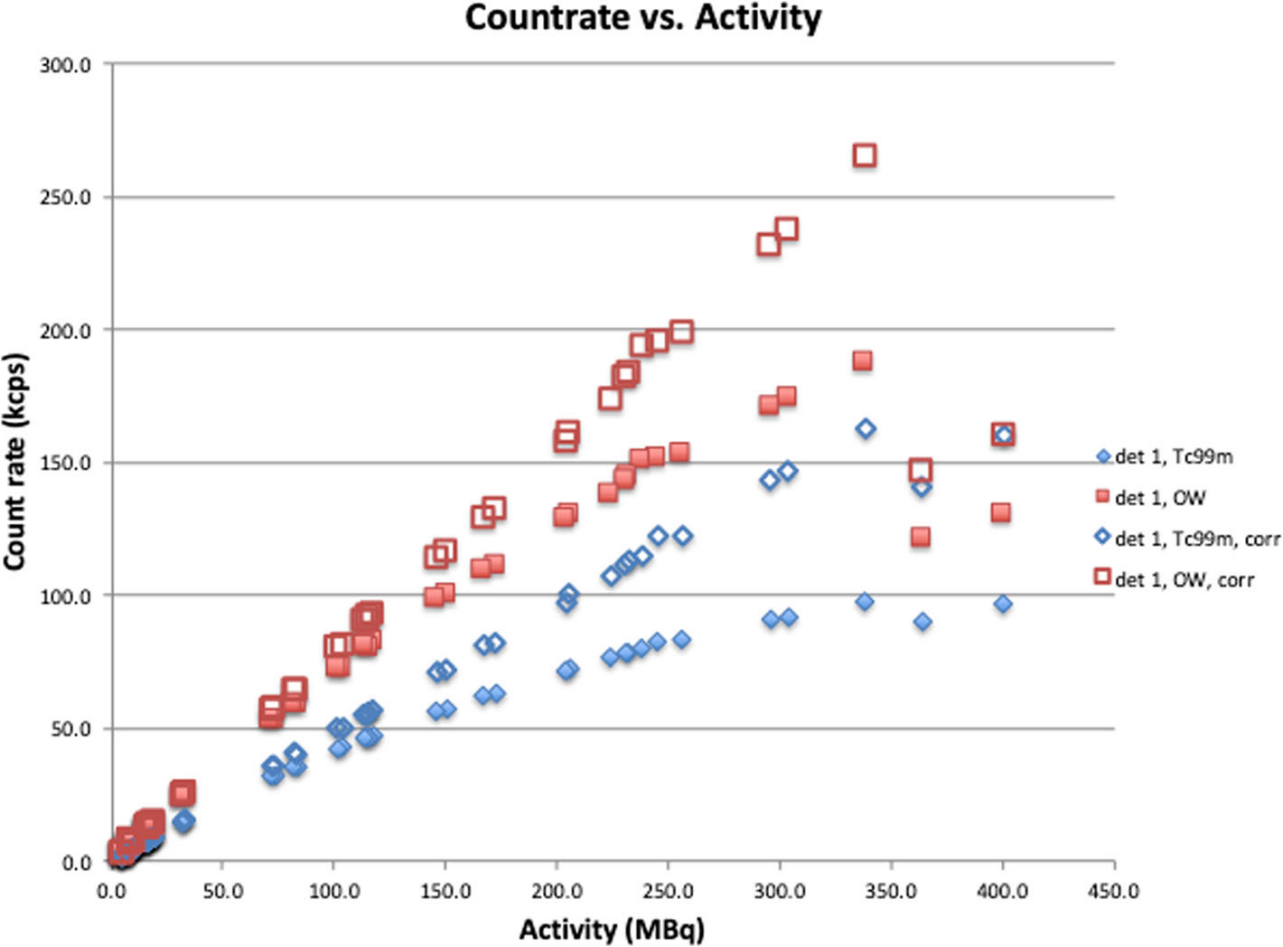
Lambert dead time corrected count rates vs. activity. Measured and corrected count rates (kcps) vs. *A* (MBq), based on Eq. (3), using τ values from Table 2, for the intrinsic measurement for detector 1 for both energy windows: OW and ^99m^Tc. The slope of the corrected curve equals sensitivity, *C*

Intrinsic sensitivity, *C*_EW_, for both detectors (kcps/MBq) and standard deviation for both energy window settings, from the graphical analysis and from the corrected count rate fit based on the dual source method (Fig. 4)

### Lambert dead time correction

**Fig. 5:**
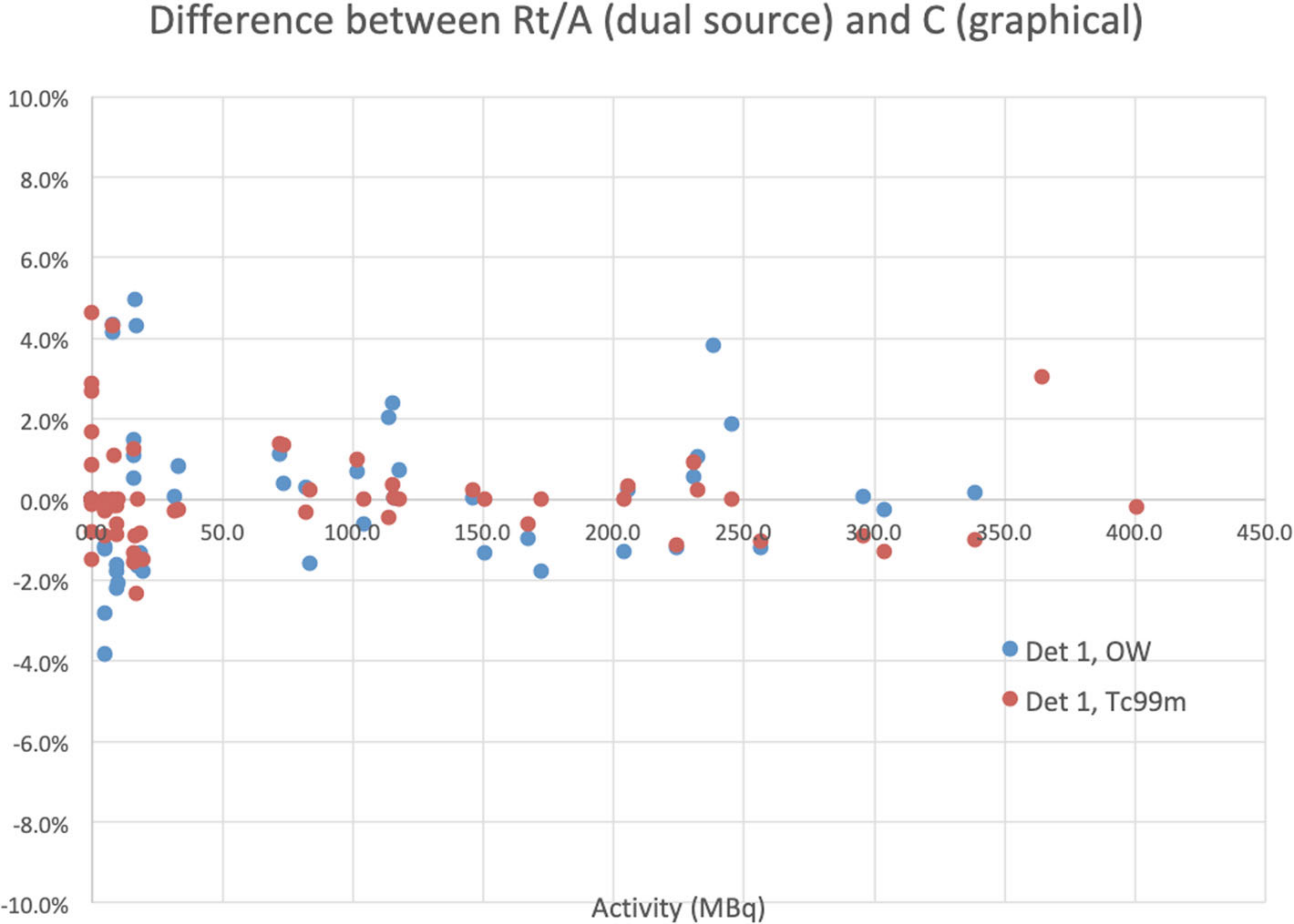
Comparison of the dual source method and the graphical analysis. Difference between the sensitivity, *C* (i.e., *R*_*t*_/*A*), estimated from count rates corrected with the dual source method, and *C* from the graphical analysis. The difference is plotted against activity, for the intrinsic measurements. Two low-activity measurements with presumed background (12% and 25% overestimation of activity) and the two points where PDM does not apply (both 49% underestimation) are outside the *y*-range of this plot.

Applying the calculated values for τ in the Lambert correction formula, Eq. (3), provides corrected count rates, *R*_*t*_*.* The results for the dual source values are shown in Fig. 4. The accuracy of a linear fit of *R*_*t*_ vs. activity (*R*^2^ > 0.999, when excluding the data points above 350 MBq) demonstrates that the dead time correction accurately restores the linear behavior in Eq. (1), with slope equal to the sensitivity, *C*_EW_. Table 3 below lists the estimated sensitivity and its standard deviation for both detectors and both energy windows, comparing it to *C* calculated from the graphical analysis. Figure 5 shows the difference between the *C* values from the graphical and dual source methods, expressed as a percentage of the graphical value.

Figures 4 and 5 and Table 3 show that the Lambert dead time correction not only restores the linear behavior of Eq. (1), but furthermore, that the sensitivity calculated from the dead time corrected count rates is very close to that calculated from the graphical analysis.

### Extrinsic measurements (LEHR collimators) with point sources

**Fig. 6:**
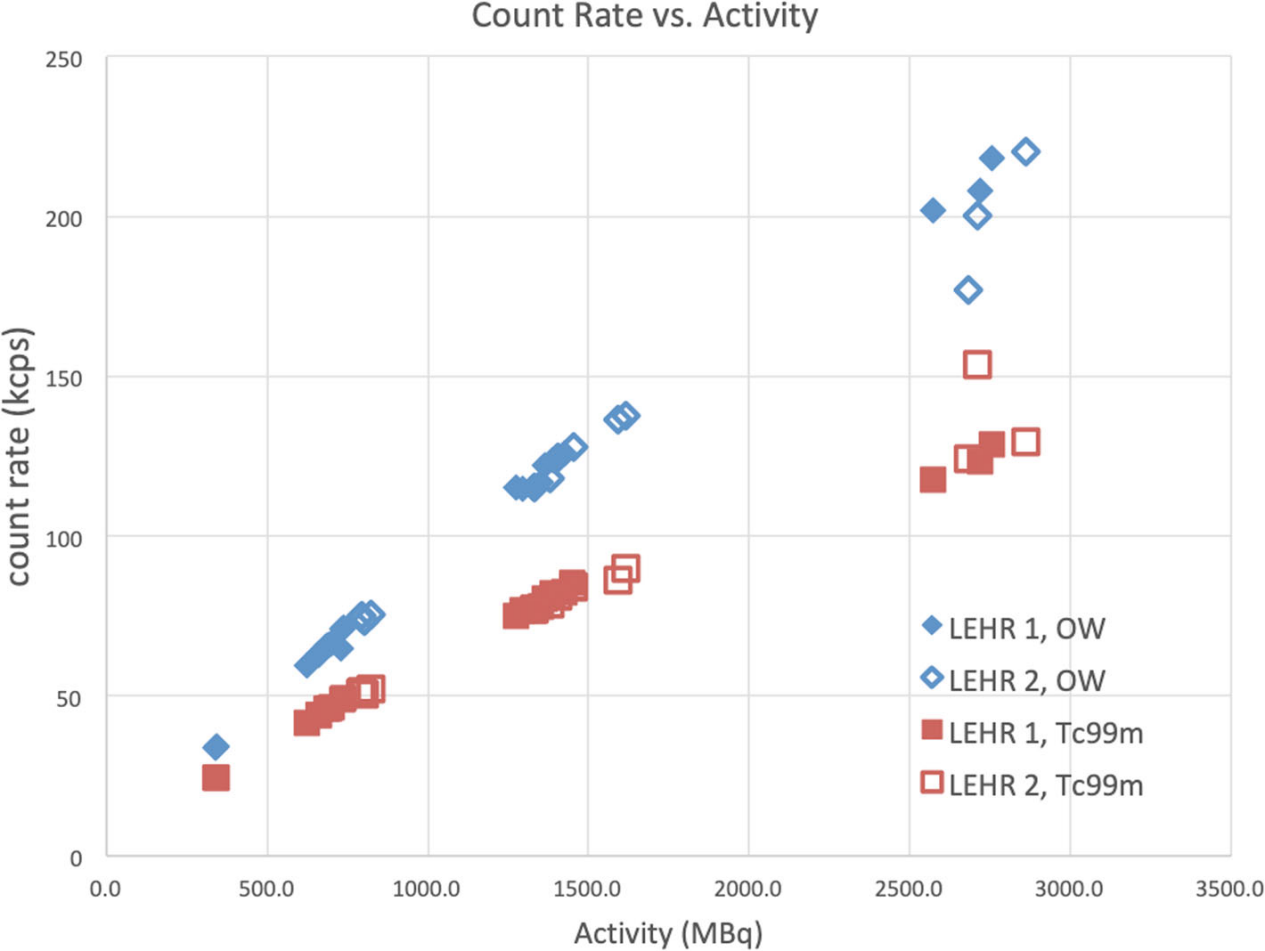
Extrinsic count rate measurement with point source. Measured count rates, *R*′ (kcps), vs. *A* (MBq) for extrinsic (LEHR collimator) measurements of a point source. LEHR 1 refers to the first, LEHR 2 to the second measurement, of detector 2 for both energy windows: OW (blue diamonds) and ^99m^Tc (red squares)

Figure 6 shows the measured count rates (*R′*) vs. activity for the extrinsic measurements with point source placed in air between the detectors. The dead time values calculated with the graphical method are listed in Table 4. These dead time values are somewhat lower than those measured intrinsically (i.e., without collimators) for both energy windows.

**Table 4:** Dead time values τ_EW_ (μs) determined using the graphical method

Detector	Energy window	Intrinsic τ (μs)	Point source 1 τ (μs)	Point source 2 τ (μs)	Scatter phantom 1 τ (μs)	Scatter phantom 2 τ (μs)
Det 1	OW	1.30	1.05	1.07	0.92	0.94
	^99m^Tc	3.16	2.69	2.61	3.6	4.29
Det 2	OW	1.25	1.01	0.99	0.87	0.93
	^99m^Tc	3.32	2.33	2.57	3.58	4.44

Dead time values τ_EW_ (μs), as determined from the intrinsic and the extrinsic (LEHR, with NEMA scatter phantom) measurements, using the graphical method

### Extrinsic measurements (LEHR collimators) with NEMA scatter phantom

**Fig. 7:**
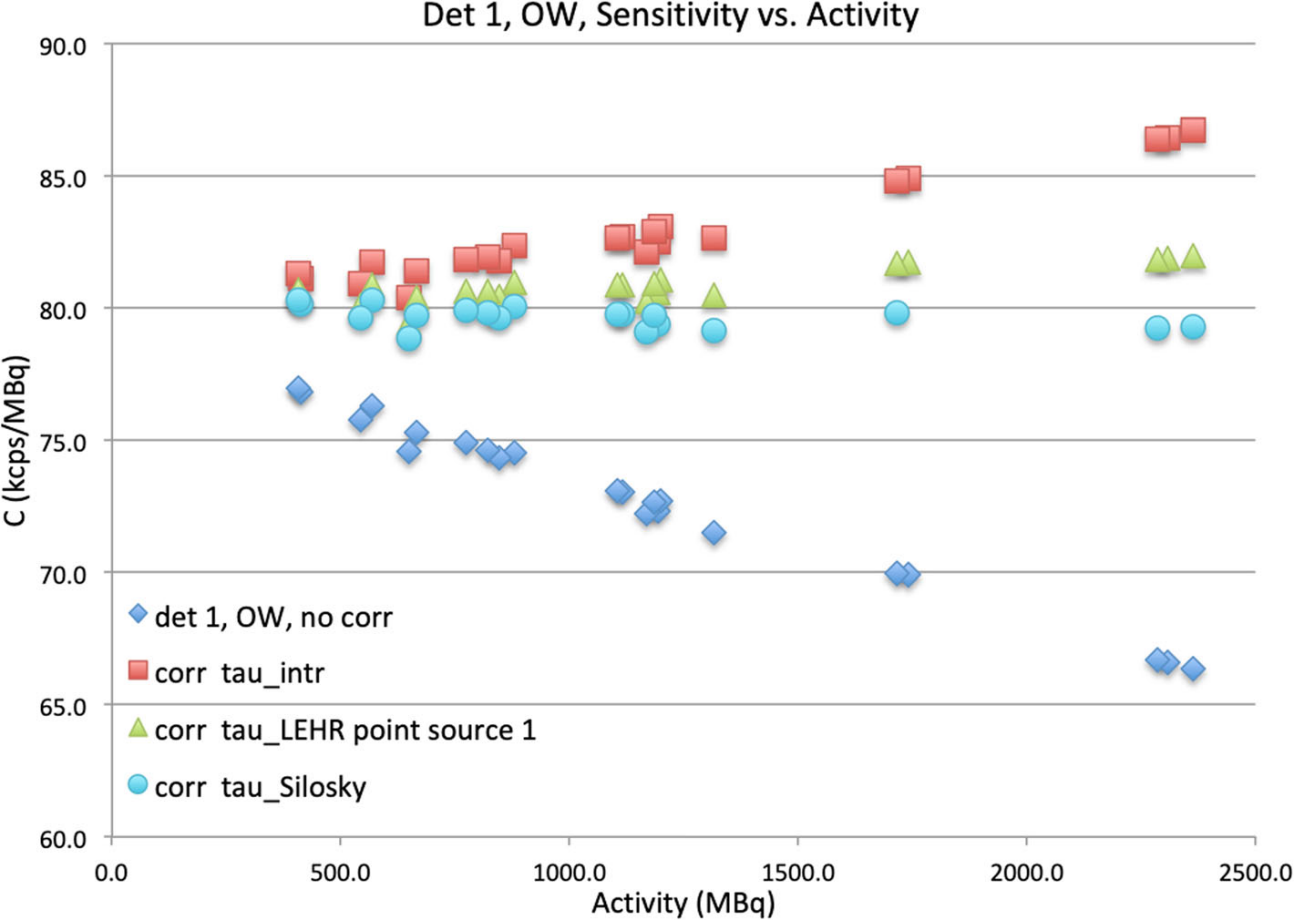
Dead time corrections for the OW measurement of the phantom. Sensitivity (*C*_OW_) in kcps/MBq vs. *A* (MBq) for the second extrinsic phantom measurement, detector 1, open window, with values of τ_OW_ from different measurements used for correction: blue diamonds, uncorrected; red squares, τ_OW_ from the intrinsic point source measurements (from Table 2); green triangles, τ_OW_ from the first extrinsic point source measurement (Table 4); and cyan circles, τ value of Silosky [3] (based on MCR).

**Fig. 8:**
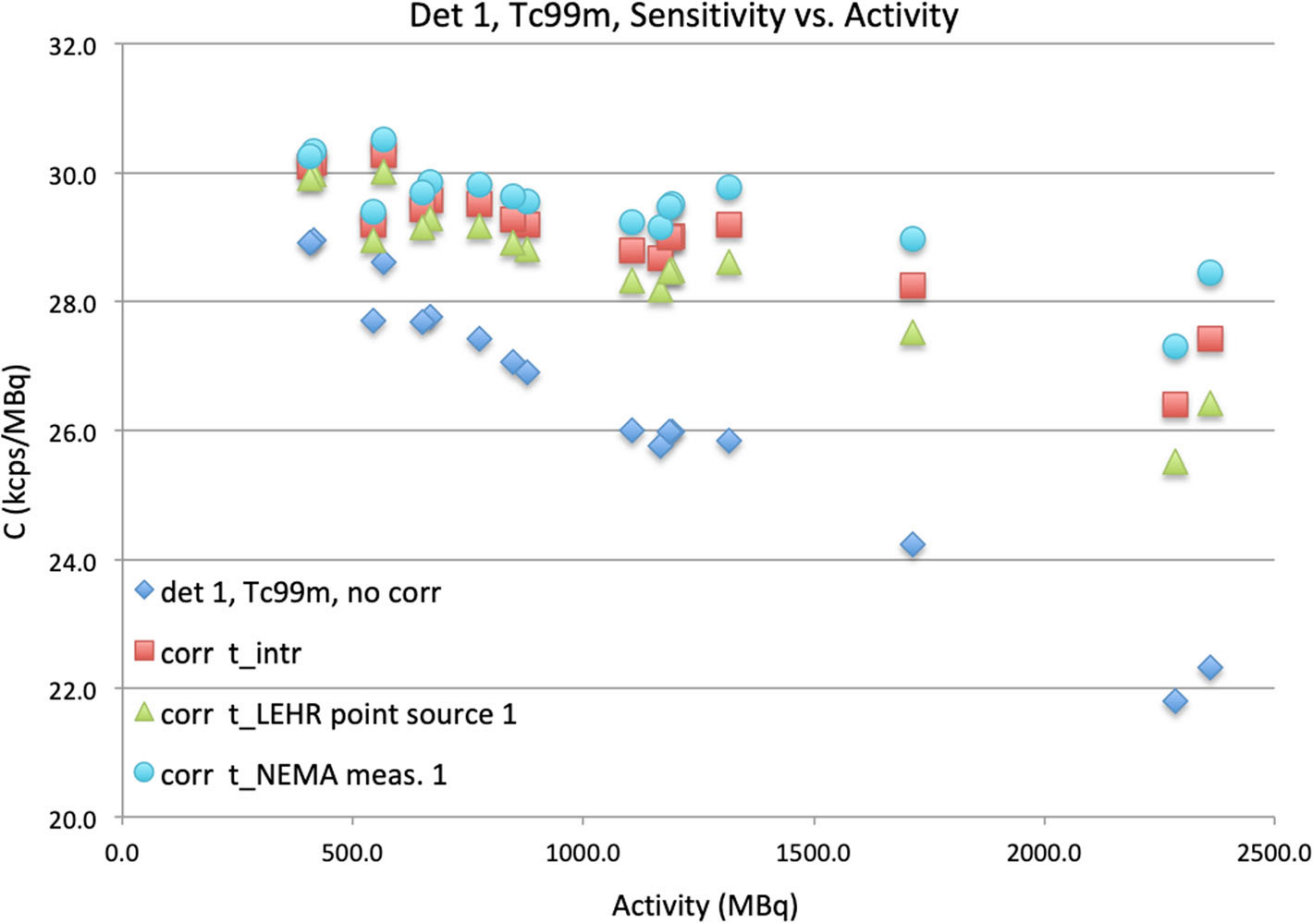
Dead time corrections for the ^99m^Tc window measurement of the phantom. Sensitivity (*C*_Tc_) in kcps/MBq vs. *A* (MBq) for the second extrinsic phantom measurement, detector 1, ^99m^Tc energy window. Dead time values τ_Tc_ (i.e., from photopeak windows) from different measurements were used for correction: blue diamonds, uncorrected; red squares, τ_Tc_ measured in the intrinsic point source measurements (from Table 2); green triangles, τ_Tc_ from the first extrinsic point sources measurements (Table 4); and cyan circles, τ_Tc_ from the first scatter phantom measurement (also Table 4)

The data of the scatter phantom measurement were corrected using the Lambert *W* function, Eq. (4), using the values of τ determined above (Table 4, point source column). The results of these corrections are plotted in Fig. 7 for the OW and in Fig. 8 for the ^99m^Tc window. For clarity, in Figs. 7 and 8, the count rate values (*R*′ or *R*) have been divided by activity, *A*; an accurate correction should result in a proportionality constant, *C*, being constant vs. *A*.

For both the open and ^99m^Tc windows, we only plot the correction using τ values from the first extrinsic point source measurements, because the τ values from the second extrinsic point source measurements gave nearly identical results (< 0.4% difference for any corrected count rate).

Figure 7 demonstrates how the accuracy of the correction was dependent on the value of τ that was used, with the extrinsic τ_OW_ values (measured with point sources, without scatter phantom) improving over the intrinsically measured τ_OW_. However, the τ_OW_ value that best corrected the count rate loss was that determined for the same type of scanner in Silosky et al. [3], with a value of 0.94 μs, based on the maximum count rate (MCR), averaged over both detectors.

From these extrinsic scatter phantom measurements, values for an effective dead time τ_EW_ were determined as well. These are also listed in the last two columns of Table 4, below.

The value of τ_OW_, measured from the extrinsic scatter phantom measurement with the open energy window, was for both detectors approximately 30% lower than that determined from the intrinsic measurement, while it corresponded quite well with the extrinsic point source measurement (< 15%). Similarly, the value that Silosky et al. [3] have measured from the maximum count rate, 0.94 μs, corresponded very closely.

For a final verification of the dead time calculation and correction methods, we used the τ value from the first scatter phantom measurement series to correct the second series. For the open energy window measurements, corrections for detectors 1 and 2 were accurate to 0.4% (within standard deviation) and 0.8%, resp., but both slightly underestimated.

As can be seen from Fig. 8, however, none of the measured τ_Tc_ values sufficiently corrected the second extrinsic NEMA phantom measurement.

### Effect of energy window settings on dead time

For the intrinsic measurements, OW and ^99m^Tc energy windows were measured simultaneously, and for the extrinsic measurements, 4 additional energy windows (14 keV wide) were measured (also simultaneously) at 85%, 95%, 105%, and 115% of the ^99m^Tc photopeak. These last energy windows were summed to create a 40% wide (112.4–168.6 keV) energy window as well.

**Fig. 9:**
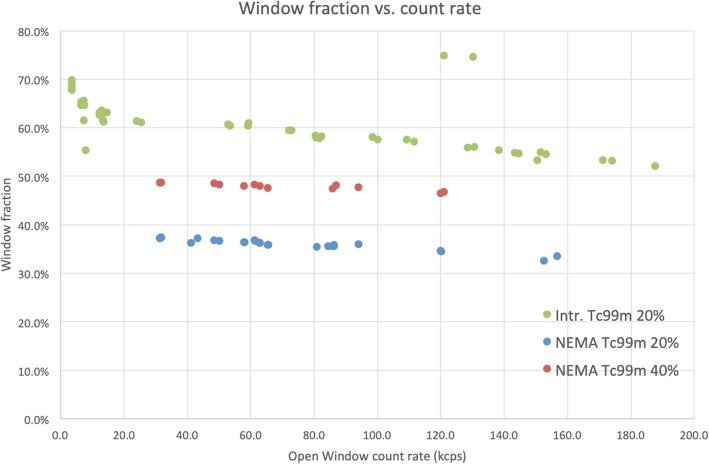
Window fraction (i.e., ^99m^Tc counts/OW counts) plotted vs. open window count rate for the intrinsic measurement and the second extrinsic measurement with the NEMA scatter phantom for both 20% and 40% ^99m^Tc energy window width. Green, intrinsic measurement 20% ^99m^Tc energy window; blue, NEMA phantom measurement 20% ^99m^Tc energy window; red, LEHR NEMA phantom measurement 40% ^99m^Tc energy window. The 40% energy ^99m^Tc window is not available for all measurements

Figure 9 shows the window fraction, i.e., ^99m^Tc energy window counts/OW counts, for the intrinsic measurement (20% ^99m^Tc window only) and an extrinsic measurement (20% and 40% ^99m^Tc energy windows) for the NEMA scatter phantom. Very similar curves were acquired for the extrinsic point source measurement in air. Note that the two outlying points for the intrinsic measurement (green) in Fig. 9 were measured at higher activity, where the OW count rate experienced a degradation (see Fig. 1) both increasing the ^99m^Tc window fraction, as well as simultaneously shifting the data points to the left on the horizontal axis.

As can be seen from Fig. 9, the window fraction, both for the 20% and 40% windows, decreased almost linearly with count rate. This was the case for all measurements; Table 5 shows the percentage loss of window fraction for each of the measurements. The loss is listed as a percentage decrease in window fraction per 100 kcps count rate, calculated as the slope of a unweighted least squares linear fit of the window fraction, over the entire open window count rate range, multiplied by 100.

**Table 5:** Percentage loss of energy window fraction per 100kcps increase in count rate

	Energy window	Intrinsic (%)	Point source 1 (%)	Point source 2 (%)	Scatter phantom 1(%)	Scatter phantom 2 (%)
Det 1	^99m^Tc (20%)	− 9.5	− 9.4	− 7.5	− 5.1	− 8.4
	^99m^Tc (40%)		− 6.9	− 3.5	− 3.5	− 4.6
Det 2	^99m^Tc (20%)	− 7.7	− 8.9	− 9.7	− 5.7	− 9.5
	^99m^Tc (40%)		− 6.6	− 7.1	− 3.9	− 4.5

Percentage loss of energy window fraction, for the 20% and 40% ^99m^Tc energy windows, as determined from plots of energy window fraction vs. open window count rate. The loss is expressed in a percentage per 100 kcps increase in count rate

## Discussion

Accurate quantification of NM images in a therapeutic setting requires an accurate correction of dead time losses. In the present paper, we demonstrate both a revised method to quantify the effect of dead time as well as a novel method to correct for it.

As can be seen from Figs. 1 and 4, and as has been noted by Silosky et al*.*, the paralyzable detector model breaks down above a particular count rate. Above this count rate, there is a sharp reduction in proportionality constant, whereas the photopeak window fraction increases significantly (Figs. 1 and 9). Although the Lambert dead time correction method is accurate for the paralyzable range, it could be important to discuss internal data processing with the manufacturers to better understand possible limitations of the PDM and the Lambert correction method. Interestingly, Seret [21] has observed a jump in the response of four gamma cameras of the same type as studied here, and Gregory et al. [22] mention a “fast” mode for high count rates in SPECT cameras from two other major vendors.

### Graphical method for dead time calculation

As a first step, we have validated the revised graphical method in a comparison with the dual source method, comparing both the dead time value, τ, itself, as well as the sensitivity, *C*, for an open window as well as a ^99m^Tc energy window. For the graphical method, *C* can be determined from non-dead time corrected count rates; this was compared to the value of *C* that follows from the corrected count rates from the dual source method.

As can be seen from Tables 2 and 3, the estimates of τ and *C* were very reproducible; however, we believe that the graphical method may have some advantages over the dual source method. The most important advantage is that all data points are used simultaneously by the graphical method, leading to a single value of τ. Outlying data points, e.g., where the PDM is no longer applicable, or where background is not sufficiently corrected, can be readily excluded from the fit. For the dual source method, a count rate range was selected (in Fig. 2) for which τ was considered constant (following [3]), even though a decrease of τ over that range might be discernable, as was found by other authors [3, 7]. Therefore, in contrast to the graphical method, the dual source method does not result in a unique value for τ. Even though in principle the dual source method would allow a determination of τ through a small number (three) of measurements, in practice, this could lead to significant errors. For instance, in Fig. 2, at low count rates, large deviations were found. These are believed to be the result of variations in background between the different images of a single dual source measurement, due to the clinical activities on other cameras in our department.

The reproducibility of the measured dead time values was examined by repeating both extrinsic measurements on two different days. The values of τ estimated for the open energy window and for the ^99m^Tc energy window were reproducible for the point sources placed in air. The reproducibility was poor however (differences exceeding 20% for the ^99m^Tc energy window) when the sources were placed in the scatter phantom (Table 4). This may be the result of inadvertent variations in the positioning of the photopeak or in the placement of the sources (which could result in different scatter counts), but this conjecture remains to be verified.

A possible explanation of the variability of τ between the intrinsic and the extrinsic measurements for the open window measurements could be an internal lower threshold for the detection of events in the detector, which nevertheless requires response time. Removal of the collimators for the intrinsic measurement drastically increases background, in particular, at the lower end of the energy spectrum. The lowest detected photon energy in the spectrum was 20 keV, cutting off a part of the spectrum. Manufacturers’ input on the inner workings of detectors might be of valuable assistance to determine the actual system value of τ.

### Lambert *W* function for dead time correction

Figures 4, 5, and 7 show that the closed form Lambert dead time correction accurately rectified the measured count rate, resulting in the linear relationship between activity and count rate.

However, one must be aware that it is in principle impossible to estimate the activity *A* from measurements above a true count rate τ^−1^, or measured count rate (*e*τ)^−1^, because Eq. (3) has two solutions for each *R*′. For the system that was investigated, the maximum count rate is not limited by the theoretical value of (*e*τ)^−1^ but by some other effect within the gamma detector. This can be seen from Figs. 1 and 4: for the open window, the dead time of 1.3 μs corresponds to a theoretical maximum true count rate of around 280 kcps, whereas the model (and subsequently the correction method) breaks down at a much lower count rate.

When measuring a single value of *R*′, as is the case for patient imaging, it is impossible to determine if one is above or below maximum count rate, *R*′_max_. This will affect any dead time correction method except possibly one where a calibration source is simultaneously measured in the image (e.g., [11]). It is therefore important to determine the count range for which PDM applies and to verify that clinical applications remain well within that range.

Even though the Lambert dead time correction is in principle exact, the accuracy of the correction is dependent on the value of τ that is used. An important issue is whether dead time values estimated intrinsically or extrinsically with point sources in air can be used to restore linearity with clinical data, within an appropriate activity range. For the scatter phantom measurements, as shown by Fig. 7, good linearity was obtained for the open window despite somewhat larger deviations when using the intrinsic value of τ_OW_. The extrinsically measured τ_OW_ for point sources in air restored linearity to within 5%. For the ^99m^Tc energy window, however, it seems that the τ_Tc_ values determined with point sources in air, either intrinsically or extrinsically, were not accurate enough for correction of the scatter phantom data.

### Effect of energy window settings on dead time

The use of a dead time parameter, τ, based on ^99m^Tc window data only, does not reflect the fact that the dead time is caused not only by the photons in the acquired energy window, but by *all* photons detected by the camera.

However, even though Adams et al. [7] and Silosky et al. [3] show a close to inverse relationship between τ and the photopeak fraction, Uribe et al. [5] report that Eq. (6) did not correctly predict any of the dead time values they measured, using values of η ranging from 1 to 1.4 (as reported in literature). Therefore, they do not support its use.

In the present paper, too, we found from Tables 2 and 3, e.g., for detector 1, that *C*_Tc_ ∙ *τ*_Tc_ = 1.53 was much larger than *C*_OW_ ∙ *τ*_OW_ = 1.04. For the intrinsic measurements, Eq. (6) would require a value for η of 1.83. The fact that τ_Tc_ was larger than expected implies more ^99m^Tc photons were lost with increasing count rate.

This loss of ^99m^Tc events is most probably the result of pulse pile-up, which causes a broadening of the ^99m^Tc photopeak, resulting in a decrease of photopeak fraction with increasing count rate for the pre-defined energy window (140 keV ± 10%). This broadening has been observed for example by Lewellen and Murano [17] and is also clearly apparent from Fig. 9 and Table 5.^4^ The impossibility to define a reliable value of τ for a fixed energy window is clearly demonstrated by the fact that, for instance, the 155–168 keV window in our experiments showed a negative value for τ, which is of course not physical.

A second consequence of Eq. (6), regardless of the value of η, is that an accurate determination of τ for the relevant energy window requires a window fraction *w*_*f*_ for the calibration setup close to that of a clinical scan. Because the window fraction largely depends on the scatter properties of the imaged object, we observed differences larger than 50% between the dead times estimated from the ^99m^Tc data with point sources in air and in the NEMA phantom. We believe that the application of a single (intrinsically measured) τ_Tc_ for extrinsic phantom or patient measurements is not advisable.

### Suggested dead time correction method

An optimal quantification of the activity in nuclear imaging requires complex methods relying on Monte Carlo simulations [23] to accurately model the object and the acquisition electronics. Nevertheless, the simple paralyzable model with the Lambert dead time correction is practical and allows a significant and robust improvement of the linearity, as shown for instance by Fig. 7.

We suggest to correct for dead time and pulse pile-up separately, using a *detector* value of τ, τ_OW_, in combination with a correction for the decrease of window fraction with (open window) count rate. This could be implemented through the use of list mode data, perhaps with quasi real-time information on dead time, or with energy window settings based on actual energy resolution, which is known to decrease with count rate [17, 23]. In the most straightforward setting, window fraction could be determined by measuring open window and photopeak window simultaneously (as has been suggested in [19]).

In the experiments presented here, we have demonstrated the correction method in a simple model: a commonly used (and readily available) isotope, ^99m^Tc, with a single photopeak. For future investigations, we want to evaluate other isotopes such as ^131^I and ^177^Lu, firstly, because these isotopes are used at high activities and count rates in therapeutic imaging, and secondly, to replicate our findings for gamma spectra comprising multiple photopeaks and increased scatter. With regard to dead time effects within the ^99m^Tc energy window, for instance, we have already found that correct peaking of the signal is an absolute necessity for accurate measurements.

Additional investigations will also be needed to verify the validity of the proposed dead time correction method in planar or SPECT imaging.

Because the proposed correction method is based on the open window, encompassing the entire spectrum, we are confident that given an accurate value of the dead time parameter τ, the method presented here can provide a straightforward and robust tool for dead time correction for quantitative nuclear imaging, even in cases of multiple photopeaks and increased scatter levels, such as for ^131^I and ^177^Lu.

## Conclusion

In the present paper, we have demonstrated a straightforward analytic dead time correction based on the Lambert *W* function. The expression for correcting these dead time losses is exact for the range of activities for which the paralyzable detector model applies. However, its accuracy is dependent on the accuracy of the dead time parameter τ.

For the estimation of this single parameter τ, we have implemented a revised “graphical” method for fitting the count rate of decaying sources. However, investigation of the dependence of dead time losses on experimental setup and energy window settings indicates that measured τ values are variable with geometry as well as window fraction.

The closed form expression for dead time correction based on the Lambert *W* function has to our knowledge not been used in nuclear medicine.

## Data Availability

The datasets acquired and analyzed for this manuscript are available from the corresponding author on reasonable request.

## Abbreviations

NM: Nuclear medicine
TRT: Targeted radionuclide therapy
ROI: Region of interest
MIRD: Committee on Medical Internal Radiation Dose
EW: Energy window
SPECT: Single photon emission computed tomography
PDM: Paralyzable detector model
NEMA: National Electrical Manufacturers Association
UFOV: Uniform field of view
OW: Open window
PET: Positron emission tomography
LEHR: Low-energy high-resolution (collimators)
MCR: Maximum count rate

## Footnotes

1 Eq. (1) is taken from [1], where background is expressed in a multiplicative i.o. an additive manner. In the following, we correct measured count rates for background (which, for point sources, is not due to activity elsewhere) by simple subtraction and set *F* = 1.

2 The formula is originally from Evans RD: The atomic nucleus New York, McGraw-Hill, 1955, pp. 785–788.

3 Silosky et al. [3] present another method for analyzing dead time that does not neglect dead time losses at low count rate nor does it require background measurements. However, in part because of the complexity of the model to be fitted, this *counts method* was not considered in the present paper.

4 This effect is also discussed in the context of quantitative ^177^Lu SPECT in MIRD pamphlet 26 [19].
